# Clot patterns determined by DSA and CTA can help predict intracranial atherosclerotic stenosis in acute ischemic stroke patients

**DOI:** 10.3389/fneur.2024.1395764

**Published:** 2024-07-24

**Authors:** Jichuan Li, Jianhong Yang, Xiang Gao, Qing Han, Yuefei Wu, Qing Shang, Yueshi Huang, Yao Xu, Yi Huang, Longting Lin

**Affiliations:** ^1^Department of Neurology, The First Affiliated Hospital of Ningbo University, Ningbo, Zhejiang, China; ^2^Department of Neurology, The First People's Hospital of Yuexi County, Liangshan, China; ^3^Department of Neurosurgery, The First Affiliated Hospital of Ningbo University, Ningbo, Zhejiang, China; ^4^Key Laboratory of Precision Medicine for Atherosclerotic Diseases of Zhejiang Province, Ningbo, China; ^5^Sydney Brain Center, University of New South Wales, Sydney, NSW, Australia

**Keywords:** clot pattern, intracranial atherosclerotic stenosis, stroke, digital subtraction angiography, computed tomography angiography, residual stenosis

## Abstract

**Background:**

This study examines whether clot patterns at large artery occlusion sites, as observed using digital subtraction angiography (DSA) and computed tomography angiography (CTA), can reliably indicate intracranial atherosclerotic stenosis (ICAS) in acute ischemic stroke (AIS) patients.

**Methods:**

We conducted a retrospective analysis of patients treated with stent retriever thrombectomy for intracranial occlusions at our institute since 2017, with follow-up assessments conducted at 3 months. The patients were grouped based on the initial angiography clot topographies (i.e., cut-off or tapered signs). We assessed the potential of these topographies in predicting ICAS, including a clinical outcome analysis based on clot pattern, age, Trial of Org 10172 in Acute Stroke Treatment (TOAST) classification, and onset-to-door time.

**Results:**

Among 131 patients (with a mean age of 66.6 years), the clot pattern emerged as a significant predictor of ICAS. The DSA-based model had a predictive area under the curve (AUC) of 0.745, with 55.1% sensitivity and 94.0% specificity. A multivariate model including age, onset-to-door time, TOAST classification as large artery atherosclerosis (LAA), and the presence of the tapered sign in clot patterns had an AUC of 0.916. In patients over 65 years of age with an onset-to-door time of >5 h and exhibiting a tapered sign in the clot pattern, the AUC reached 0.897. The predictive ability of the tapered sign was similar in DSA and CTA, showing 73.4% agreement between modalities.

**Conclusion:**

The clot pattern with the tapered sign as observed using DSA is significantly associated with ICAS. Incorporating this clot pattern with age, TOAST classification as LAA, and onset-to-door time enhances the prediction of ICAS. The clot pattern identified by CTA is also a reliable predictor, highlighting the importance of assessing clot patterns in ICAS identification.

## Introduction

Stent retriever (SR) thrombectomy has been a primary endovascular treatment (EVT) for acute intracranial large artery occlusion (ILO), providing a high recanalization rate and improved clinical outcomes for patients who undergo this procedure. In the REVASCAT trial, successful vessel recanalization with thrombectomy resulted in an improved clinical effect compared to the control group, with complete vessel recanalization rates of 70.5 and 22.3%, respectively (1). Similarly, the MR-CLEAN trial reported a recanalization rate of 75.4% in the group that underwent thrombectomy (2). Moreover, the proportion of patients in the endovascular treatment group with a 90-day modified Rankin Scale (mRS) score of 0–2 points was higher than in the control group [43.7% vs. 28.2%, adjusted odds ratio (OR) = 2.1, 95% confidence interval (CI) = 1.1–4.0] (1).

Intracranial atherosclerotic stenosis (ICAS) occlusion caused by atherosclerosis is a common occurrence in patients with ILO, necessitating additional medications and surgical interventions along with SR thrombectomy. ICAS occlusion can be identified by a residual fixed stenosis of >70% or a lower degree stenosis with a tendency for reocclusion or flow impairment during the procedure (3). Huang et al. (4) reported that atherosclerotic stenosis accounted for 51% of acute cerebral infarction cases in the Asian population. The pathomechanism of acute ILO is a significant factor associated with successful SR thrombectomy and positive clinical outcomes. However, SR thrombectomy is considered less effective in cases of non-embolic ILO, likely due to intracranial atherosclerotic stenosis (5–7). Among the Asian population, acute ischemic strokes resulting from intracranial large artery occlusions are frequently caused by *in situ* atherosclerotic mechanisms or cervical internal carotid artery (ICA) involvement (8, 9).

However, determining the presence of ICAS promptly poses a challenge.

Intravascular treatment of acute ICAS occlusion often requires additional empirical operations beyond standard SR thrombectomy. Single SR thrombectomy and intravascular aspiration techniques usually fail to produce satisfactory outcomes in such cases. Based on the univariate analysis, a successful SR thrombectomy outcome was found to be associated with younger age, absence of hypertension, anterior circulation occlusion, atrial fibrillation (AF), hyperdense artery sign (HAS), and CTA-determined branching-site occlusion (10). Early identification of ICAS allows for prompt interventions, such as administration of drugs (e.g., tirofiban) and preparation of stents or drug balloons, thereby reducing the recanalization time, increasing thrombectomy success rates, and decreasing reocclusion rates.

Inconsistent conclusions regarding the prognosis of endovascular treatment for ICAS-related occlusion has been derived by previous literature. Compared with embolic occlusion, ICAS occlusion is associated with the following factors: (1) lower initial severity and younger patients (11), (2) lower success rates of SR thrombectomy, (3) higher risks of residual stenosis and reocclusion, and (4) poorer clinical outcomes (12).

Therefore, in the present study, we retrospectively analyzed clot patterns determined by digital subtraction angiography (DSA) and CTA with the aim to elucidate whether these clot patterns, along with other pertinent clinical features, can predict ICAS.

## Materials and methods

We conducted a review of consecutive patients who had acute ischemic stroke and acute ILO and underwent EVT at the First Affiliated Hospital of Ningbo University, Ningbo, Zhejiang, China since 2017. The study population comprised consecutive patients aged 18 years or older who received EVT for acute ischemic stroke caused by intracranial and/or extracranial large vessel occlusion. The occlusions included those in the internal carotid artery (ICA), middle cerebral artery (MCA), vertebral artery, and basilar artery. In addition, we collected relevant data from the onset of symptoms to 3 months after discharge during the follow-up period.

This study was approved by the Institutional Review Board of our hospital, and the requirement of informed consent for study inclusion was waived because of the retrospective study design.

The occlusive clot pattern sign was defined as the angiographic manifestation of the occlusive site before recanalization.

The cut-off sign was characterized by a distinct line between the contrast-opacified antegrade blood flow and the non-opaque occluded downstream arteries.

**Figure 1:**
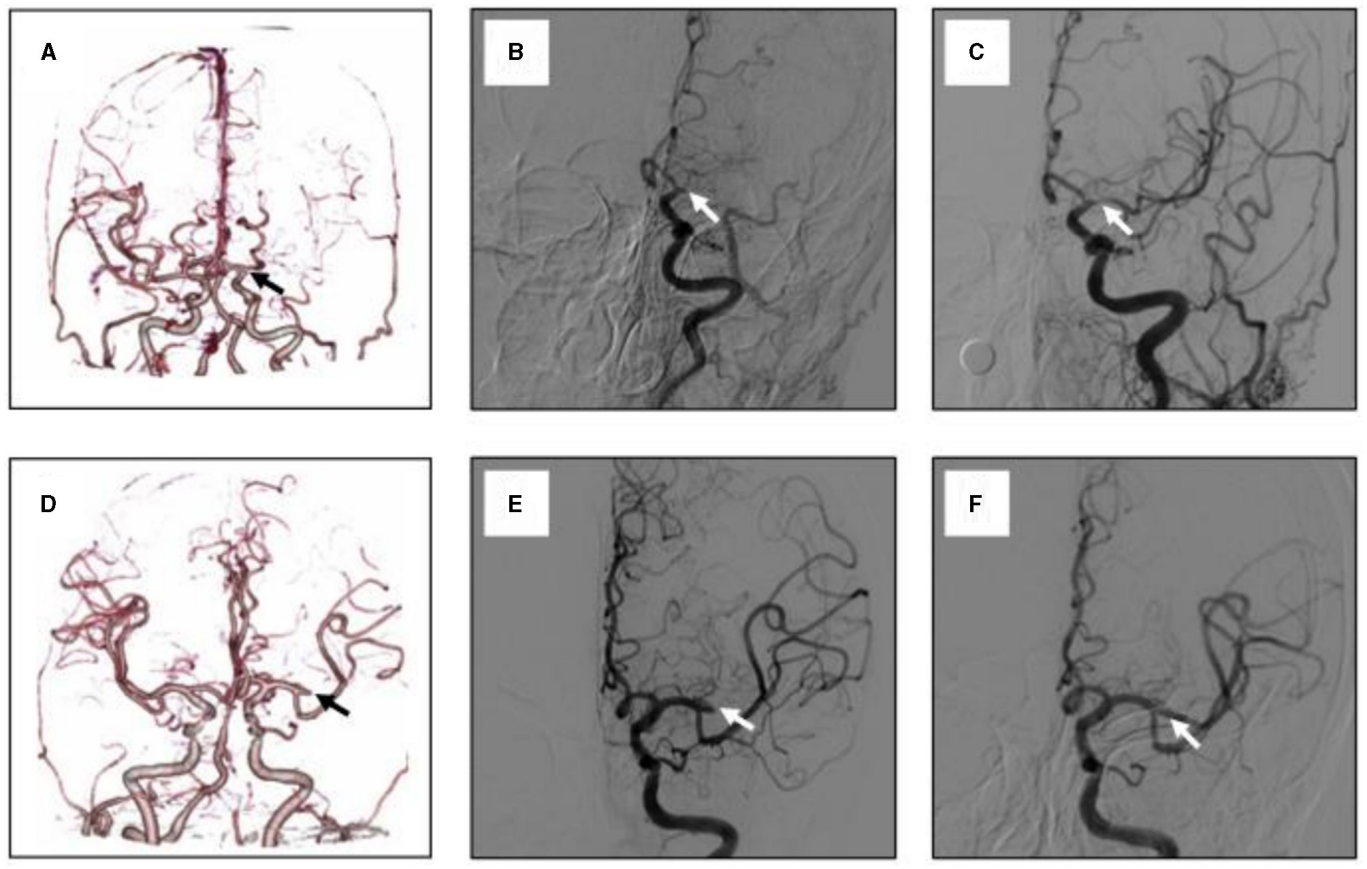
Representative cases of clot pattern at the occlusion site of MCA as visualized by CTA and DSA. **(A)** A maximum intension projection reconstruction image of CTA that showed left MCA occlusion, with the clot pattern being tapered sign. **(B)** The initial angiogram confirmed the same cite of occlusion. **(C)** The recanalization of the occluded MCA after stent retriever, the occluded MCA are recanalized, but the angiogram showed a focal stenosis of more than 50% at the M1 trunk. **(D–F)** shows another patient with left MCA occlusion. **(D)** with clot pattern being cut-off sign. **(E)** The initial angiogram also confirmed the same cite of occlusion. **(F)** Successful recanalization of the occluded MCA without stenosis after SR thrombectomy.

The tapered sign was identified by a tapered beak or flame-like contrast opacification extending into the luminal filling defect, as shown in Figure 1 (13–15).

If the grading of the occlusive clot pattern sign was difficult to determine or if discrepancies arose between the two graders, a consensus was reached. In addition, the occlusive clot pattern sign was further evaluated and possibly amended by repeated angiography following EVT during admission. Consequently, both the cut-off sign and tapered sign groups were included in the analyses.

### Inclusion and exclusion criteria

The inclusion criteria for this study were as follows: (1) Ischemic stroke patients with intracranial and extracranial large vessel occlusion, including the ICA, MCA, basilar artery, and vertebral artery; (2) those having an mRS score of 0–1 point before onset; (3) those with an onset-to-puncture time of < 24 h; and (4) those for whom successful recanalization was achieved through SR thrombectomy.

The exclusion criteria for this study were as follows: (1) patients with ILO due to uncommon stroke etiology, including arterial dissection and fat embolism found during treatment; (2) those with tandem ILO caused by extracranial arterial disease; and (3) those for whom the arterial lesion status could not be reliably assessed due to either persistent occlusion or incomplete recanalization.

### Endovascular procedures

The type of EVT procedure used in this study was SR thrombectomy. The thrombus was removed using an SR, such as the Solitaire AB (Micro Therapeutics Inc. DBA Ev3 Neurovascular, USA) or Trevo (Stryker, Kalamazoo, MI, USA). We used balloon guide catheters, adjuvant lytic infusion (tirofiban or alteplase), and performed intracranial or extracranial angioplasty and/or stenting, as required.

### Statistical analysis

Comparative analyses of clot patterns, clinical characteristics, and clinical outcomes were performed between groups with and without residual stenosis, with the degree of residual stenosis being defined as >70%. Differences among the cut-off sign and tapered sign groups were analyzed using the chi-squared tests for categorical variables and the *t*-tests for continuous variables. A simple logistic regression analysis was performed to determine the predictive value of clot patterns for ICAS. A multivariate logistic regression analysis was performed to clarify the predictive value of the onset-to-door time, age, TOAST classification, and clot pattern showing a tapered sign for identifying ICAS. The receiver operating characteristic curve analyses were performed, and their area under the curve (AUC) values were compared. In addition, the sensitivity, specificity, positive predictive value, negative predictive value, and accuracy were calculated for the CTA- and DSA-determined groups, as well as for the predictive value of clot pattern signs for ICAS.

A *P-*value of < 0.05 was considered statistically significant. All statistical analyses were performed using Stata 15.

## Results

### Baseline characteristics

A total of 131 patients underwent EVT for ILO with SR thrombectomy (Table 1). The patients (with a mean age of 66.6 ± 12.8 years) were divided into two groups: the group with (23/131) and without (108/131) of residual stenosis. In terms of the location of occlusion, 45 patients (34.4%) had MCA occlusion, 61 (46.6%) had ICA occlusion, and 25 (19.0%) had basilar artery or intracranial vertebral artery occlusion. The mean National Institutes of Health Stroke Scale (NIHSS) scores were 18.5 (range, 15–25) in the group with residual stenosis and 18 (range, 15–23) in the group without residual stenosis. The mRS scores of most patients (118/131, 87%) were ≤ 2. Using the chi-squared test or *t*-test, the onset-to-door time, clot pattern, age, and TOAST classification as large artery atherosclerosis (LAA) were found to be associated with residual stenosis (*P* < 0.05).

**Table 1:** Characteristics of patients with ILO after stent retriever thrombectomy.

	**With residual stenosis**	**Without residual stenosis**	***P*-value**
Number	23	108	
Age, SD	62 (13.1)	67.5 (12.5)	0.029
Atrial fibrillation, *n* (%)	4 (17.4)	62 (57.4)	0.000
Male sex, *n* (%)	17 (73.9)	57 (53.17)	0.070
Hypertension, *n* (%)	14 (60.9)	61 (56.5)	0.699
Diabetes, *n* (%)	4 (17.4)	15 (13.9)	0.824
Smoking, *n* (%)	7 (30.4)	21 (19.4)	0.445
Hypercholesterolemia, *n* (%)	4 (17.4)	27 (25.0)	0.651
Clot pattern tapered sign, *n* (%)	13 (56.5)	6 (5.6)	0.000
Onset-to-door time (mins) median (IQR)	491.1 (320.9–661.4)	273.1 (232.2–314.1)	0.000
TOAST (LAA), *n* (%)	12 (52.2)	16 (14.8)	0.000
IV of TPA, *n* (%)	8 (34.8)	36 (33.3)	0.882
Initial NIHSS score, median [IQR]	18.5 (15–25)	18 (15–23)	
Occlusion site, *n* (%)			0.007
ICA	8 (36.4)	36 (33.3)	
MCA	5 (22.7)	56 (51.9)	
VBA	9 (40.9)	16 (14.8)	

SD, standard deviation; TOAST, Trial of Org 10172 in Acute Stroke Treatment; LAA, large vessel atherothromboembolic; IV, intravenous injection; TPA, tissue plasminogen activator; ICA, internal carotid artery; MCA, middle cerebral artery; VBA, vertebro-basilar artery.

### Clot pattern predicting ICAS

**Figure 2:**
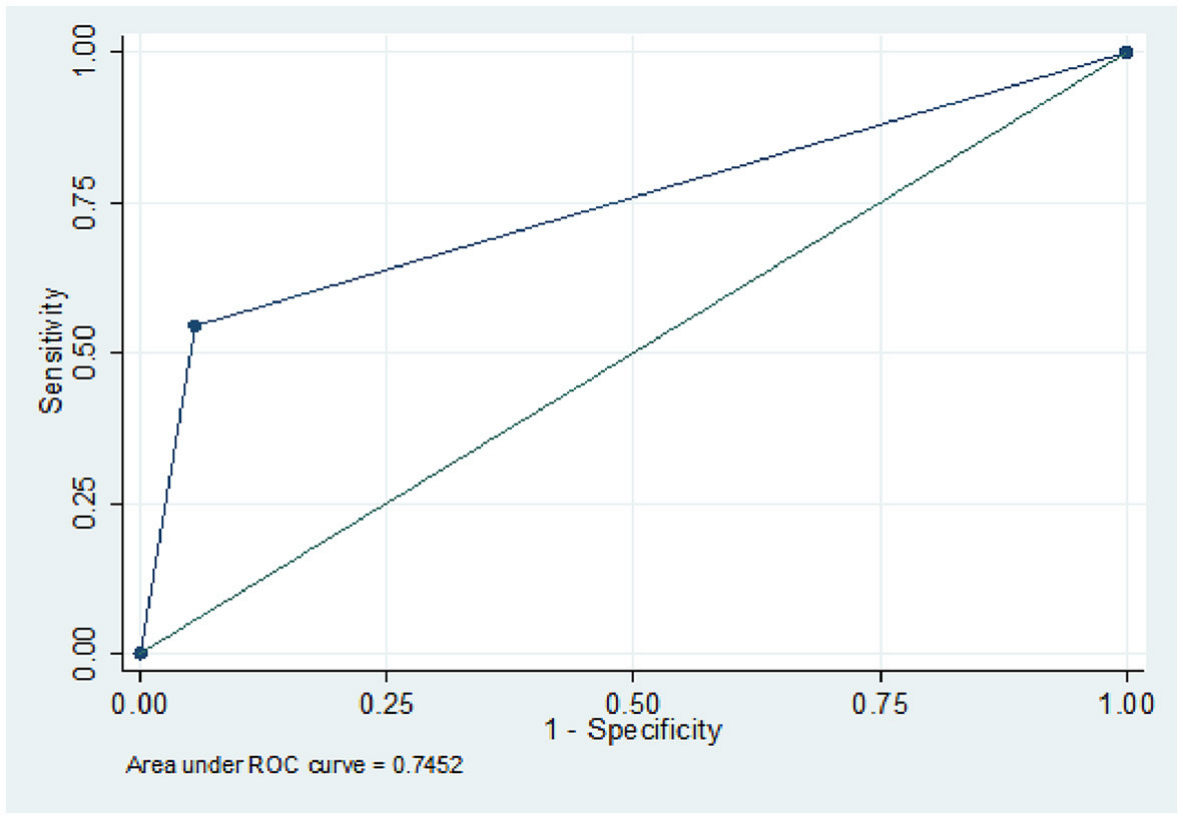
The prediction of ICAS based on clot pattern.

**Figure 3:**
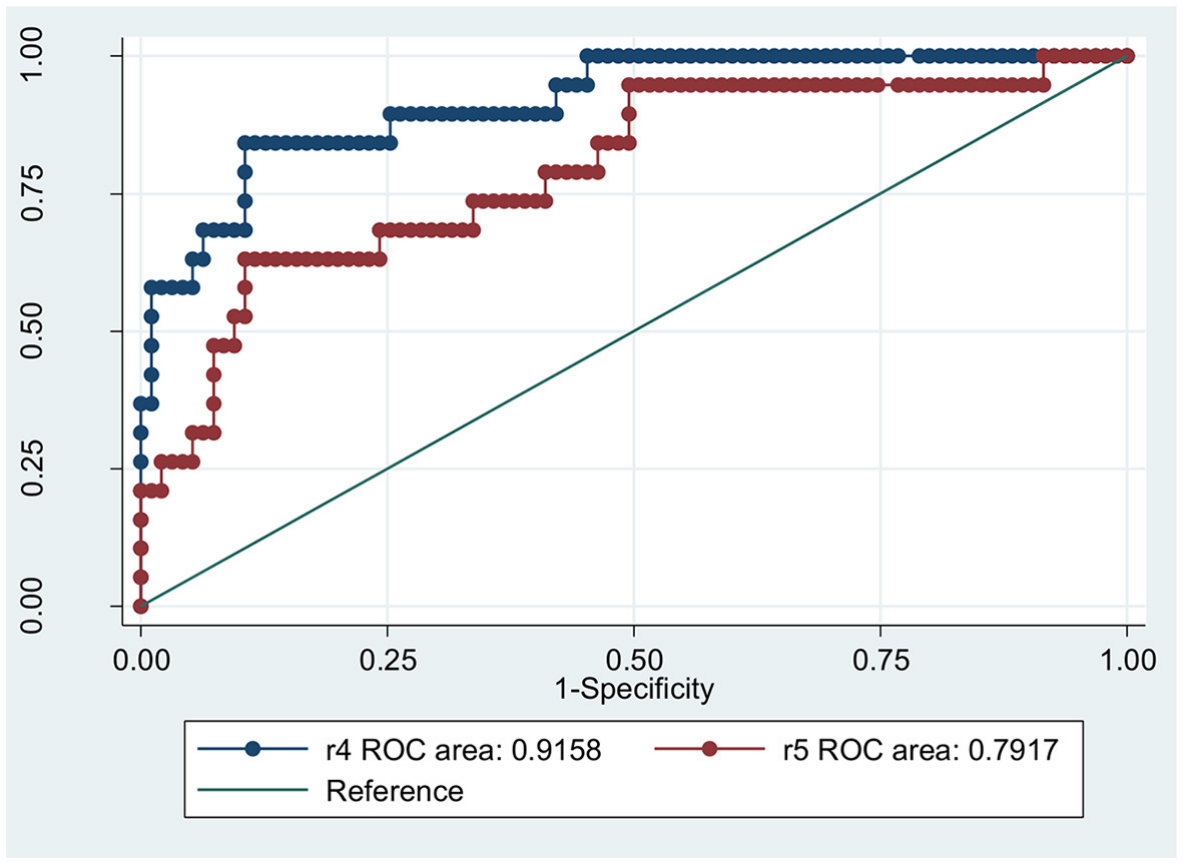
The prediction of ICAS based on clot pattern, age, and onset-to-door hour. R4 represents the combination of clot pattern, age, toast classified as LAA and onset-to-door hour, while R5 shows only age, toast classified as LAA and onset-to-door hour.

According to the results of the simple logistic regression analysis, the sensitivity and specificity of the DSA-determined clot pattern as a predictor of ICAS were 55.1% and 94.0%, respectively. The AUC based on the DSA-determined clot pattern was 0.745. These findings indicate that the clot pattern is a viable means of predicting ICAS (Figure 2). According to the results of the multivariate logistic regression analysis, age, onset-to-door time, TOAST classification as LAA, and clot pattern showing tapered signs are predictors of ICAS. The AUC based on these factors was 0.916, which suggests a high level of predictive accuracy. When clot pattern is not considered, the predictive value decreases. The AUC based on age and onset-to-door time alone was 0.792, which is lower than the AUC when the clot pattern was included (Figure 3). We used dichotomy to classify continuous variables such as age and onset-to-door time and obtained values of 64.5 and 5.3, respectively. When classifying statistics by age and onset-to-door time, patients aged more than 65 years, with an onset-to-door time of >5 h from stroke onset, TOAST classification as LAA, and clot patterns showing tapered signs are most likely to have stroke episodes caused by ICAS. The AUC for this classification was 0.897, indicating a high level of predictive accuracy (Table 2). With the onset-to-door time of >6 h instead of >5 h, the AUC value was 0.878.

**Table 2:** Results of the logistic regression model.

**Variable**	**Odds ratio (95% CI)**	***P*-value**	**AUC**
Clot pattern	27.74 (7.01–109.84)	0.000	
LAA	3.46 (0.99–12.03)	0.051	
Age >65 years	0.27 (0.08–0.97)	0.058	
Onset-to-door time >5 h	4.49 (1.23–16.34)	0.026	
Clot pattern, Age >65 years, Onset-to-door time >5 h			0.897

### Comparison of CTA- and DSA-determined clot patterns in predicting ICAS

The classification of DSA-determined occlusive clot pattern signs corresponded adequately with that of CTA-determined occlusive clot signs. Both CTA and DSA assessment results were consistent for 94 patients (73.4%, *P* = 0.059). The AUC for clot patterns determined by DSA was 0.743 (*P* < 0.001), while that for clot patterns determined by CTA was 0.732 (*P* < 0.001).

**Figure 4:**
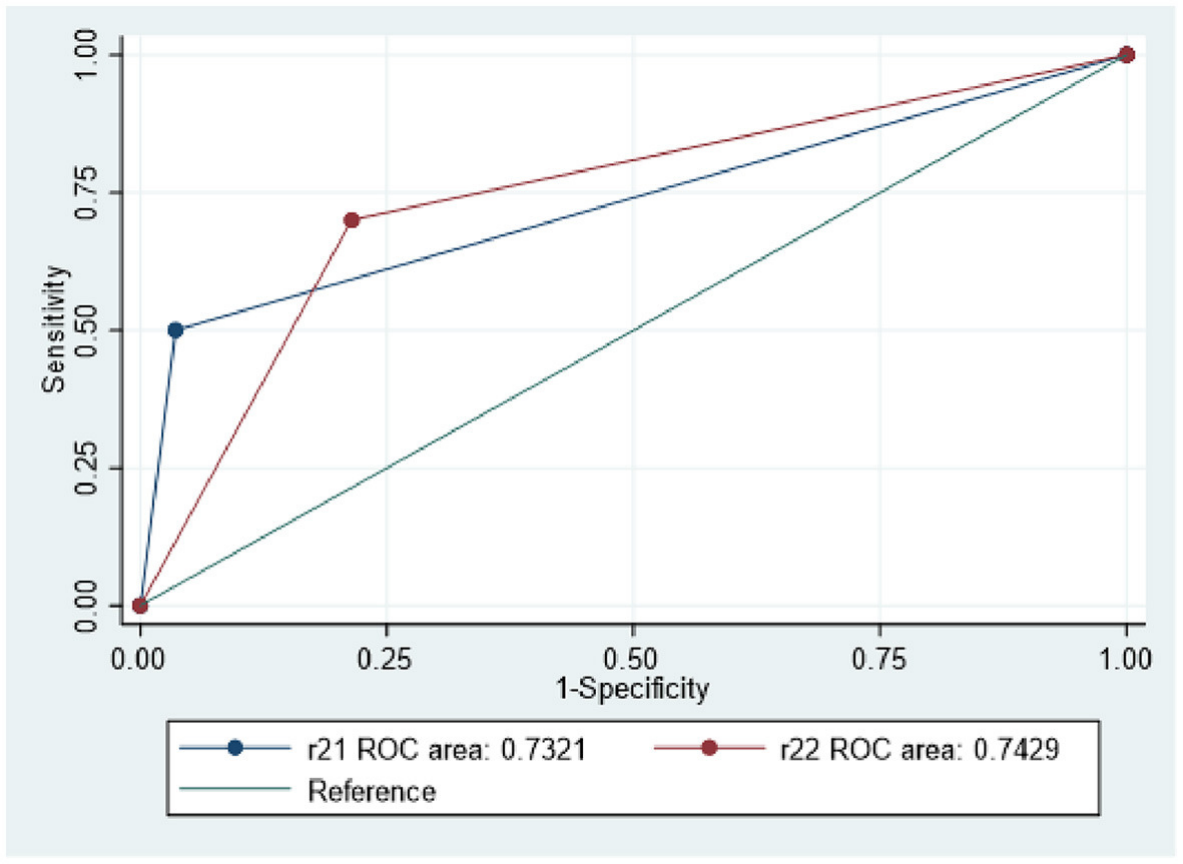
Both DSA and CTA clot pattern are predictive of ICAS. R21 depicts CTA clot pattern, while R22 represents DSA clot pattern.

The sensitivity and specificity of DSA-determined clot patterns as a predictor of ICAS were calculated to be 70 and 80%, respectively, whereas those for CTA-determined clot patterns were 50.6 and 94.9%, respectively (Figure 4). Both DSA- and CTA-determined clot patterns were capable of predicting ICAS, with DSA exhibiting greater sensitivity and CTA having preferred specificity. Therefore, depending on the clinical scenario, either of the imaging modalities may be adopted for predicting ICAS.

### Imaging and clinical outcomes after endovascular treatment

It is worth noting that, while the recanalization rates (mTICI ≥ 2b) were not significantly different between the two groups (82.14% vs. 83.33%, *P* = 0.902), there were differences in the treatment approaches used. While the stent rate was similar in both groups (3.64% vs. 6.25%, *P* = 0.617), the tapered sign group had higher rates of balloon dilation (37.5% vs. 8.18%) and tirofiban use (58.3% vs. 15.79%) compared to the cut-off sign group, suggesting that the presence of a tapered sign may prompt physicians to use these additional interventions to improve recanalization success.

No significant difference was observed in terms of intracerebral hemorrhage and death rates between the two groups. Specifically, the incidence of intracerebral hemorrhage was 38.8% (42 out of 110 patients) in the cut-off sign group and 17.7% (three out of 17 patients) in the tapered sign group (*P* = 0.099), whereas the rate of serious hemorrhagic complications was 10.9% (12 out of 110 patients) in the cut-off sign group and 11.8% (two out of 17 patients) in the tapered sign group (*P* = 0.918). These findings suggest that both groups had similar rates of hemorrhagic complications and mortality rates.

Poor outcomes, defined by an mRS score of 5 or 6 at the 3-month follow-up, were observed in 40.6 and 37.5% of patients in the cut-off sign and tapered sign groups, respectively (*P* = 0.813). Similarly, no significant difference in the death rate after 3 months was observed between the cut-off sign group (20.8%) and the tapered sign group (21.3%; *P* = 0.354). In addition, the ordinal distribution of mRS scores was insignificantly different between the two groups (*P* = 0.746, Table 3). These results suggest that the tapered sign group did not share improved functional outcomes or mortality, highlighting the need for improved treatment strategies for ICAS.

**Table 3:** Imaging and clinical outcomes following endovascular treatment.

	**Cut-off sign group (*n* = 113)**	**Tapered sign group (*n* = 18)**	***P*-value**
Recanalization, *n* (%)	92/112 (82.14)	15/18 (83.33)	0.902
Stent, *n* (%)	4/110 (3.64)	1/16 (6.25)	0.617
Angioplasty, *n* (%)	9/110 (8.18)	6/16 (37.5)	0.001
Tirofiban, *n* (%)	12/76 (15.79)	7/12 (58.33)	0.001
Good outcome at 3 month follow-up, *n* (%)	34/96 (35.42)	5/16 (31.25)	0.746
Poor outcome at 3 month follow-up, *n* (%)	39/96 (40.63)	6/16 (37.5)	0.813
Death at 3 month follow-up, *n* (%)	20/96 (20.83)	5/16 (21.25)	0.354
HT, *n* (%)	42/110 (38.18)	3/17 (17.65)	0.099
PH2, *n* (%)	12/110 (10.91)	2/17 (17.65)	0.917
SICH, *n* (%)	21/108 (19.44)	2/18 (11.11)	0.397

HT, hemorrhagic transformation; PH2, parenchymal hematoma type 2; SICH, symptomatic intracranial hemorrhage.

## Discussion

In this study, we found that clot patterns determined by both DSA and CTA can predict ICAS; however, these patterns exhibited different levels of sensitivity and specificity. In addition, considering clot patterns, along with age and onset-to-door time, in the multivariate logistic regression models improved the predictive value for ICAS. Therefore, the assessment of clot patterns can serve as a valuable approach for predicting ICAS in patients with acute ischemic stroke who undergo EVT.

Patients with ICAS commonly exhibit intraluminal filling defects, with the tapered sign being a characteristic clot pattern. This finding is consistent with prior research, which has shown tapering to be more frequently observed in the etiology of an intracranial atherosclerotic disease (14, 16). Other causes of cone or flame signs, such as extracranial ICA dissection, may obstruct distal blood flow due to factors unrelated to atherosclerosis. In addition, we observed that patients with tapered occlusion tend to present with milder symptoms on neuroimaging, smaller infarct volume, and milder neurological deficits, as well as early fluctuating symptoms in cases of super acute ischemic stroke (17). The tapered sign observed in the initial DSA can serve as a surrogate marker for ICAS-related occlusion and procedural difficulty. However, its clinical significance remains unclear (3). The initial tapered sign typically occurs in patients with acute ischemic stroke and large vessel occlusion due to ICAS. These patients may have better-established collateral circulation during the development of stenosis and possibly greater neural tissue tolerance to hypoxia.

CTA-determined clot patterns can predict ICAS earlier, with comparable prediction accuracy to DSA-determined clot patterns but with faster availability after admission. The pathogenesis of ICAS involves stenosis and acute thrombosis, resulting in perforator events and hypoperfusion. Unlike cardioembolism, early diagnosis of ICAS allows for more effective medical treatment plans, such as antithrombotic and blood pressure management. ICAS patients have similar recanalization rates but higher residual stenosis and reocclusion rates compared to cardioembolism patients. During EVT, SRs can cause intimal injury and platelet activation, leading to *in situ* thrombosis of ICAS, which may increase the risk of early reocclusion of the target vessel (17). Tirofiban, whether administered intravenously or intra-arterially, can effectively promote the dissolution of new thrombi and decrease the occurrence of reocclusion events, particularly in patients with ICAS (18). With regards to surgical options for ICAS-ILO patients, SR thrombectomy as a first-line treatment has a higher reperfusion rate and causes less vascular injury than contact aspiration (19). Furthermore, the “microcatheter first-pass effect,” proposed by Wenhuo Chen's team, helps to identify potentially stenotic areas, thereby preventing iatrogenic damage to the blood vessel wall that may result from aggressive manipulation of the intermediate catheter (20). For some patients, if our center's interventional physician determines that, balloon dilation followed by SR thrombectomy is an appropriate intervention for ICAS, it may lead to faster surgical recanalization (21).

The current study showed comparable recanalization rates between the cut-off sign and tapered sign groups. This result may be attributed to the increased use of advanced techniques, such as tirofiban, stent implantation, and balloon dilation, in the tapered sign group. In addition, both groups exhibited similar bleeding risks in this study. A recent investigation revealed that topical tirofiban infusion and angioplasty/stent therapy had similar efficacy and safe profiles in treating acute-phase ICAS occlusion. Another research demonstrated that rescue therapy with stent placement to treat underlying ICAS of the M1 segment is technically feasible in patients with AIS. However, these patients had a significantly lower rate of favorable outcomes than those with thromboembolic M1 occlusions (22). Moreover, the favorable mRS scores observed in both groups were consistent with those observed in previous studies (23).

Combining age, onset-to-door time, and clot patterns can significantly improve the predictive value for ICAS. Patients with ICAS-related occlusion typically present with lower initial NIHSS scores and milder baseline Alberta Stroke Program Early Computed Tomography Score (ASPECTS) and tend to be younger than those with embolic occlusion (12). Particularly, age can serve as a prognostic factor for good clinical outcomes following EVT. Another study found that early hospital admission (onset-to-door time ≤ 4.5 h) was significantly associated with favorable outcomes, independent of confounding factors such as reperfusion therapy and type of stroke onset (24). In our study, the multivariate model demonstrated that age, onset-to-door time, and clot patterns were consistent predictors of ICAS, while the NIHSS score indicated no significant deviation.

One limitation of this study is its small sample size, which may limit the generalizability of the findings. In addition, several factors can affect clot patterns on CTA, such as occlusion location, bone tissue interference, head motion artifacts during imaging, and variations in blood flow between the proximal and distal segments of the occluded vessel. These factors can also affect the accuracy of DSA in assessing clot patterns. Studies considering a larger sample size are needed to confirm the results of this study and to further explore the predictive value of clot patterns for ICAS.

## Conclusion

The tapered sign observed in DSA-determined clot patterns is significantly associated with ICAS. Models incorporating age, onset-to-door time, TOAST classification as LAA, and a clot pattern showing tapered signs exhibit a strong predictive value for ICAS. Furthermore, clot patterns determined by CTA have a good predictive value, with higher specificity for ICAS.

## Data availability statement

The raw data supporting the conclusions of this article will be made available by the authors, without undue reservation.

## Ethics statement

The studies involving humans were approved by the Ethics Committee of the First Affiliated Hospital of Ningbo University. The studies were conducted in accordance with the local legislation and institutional requirements. The participants provided their written informed consent to participate in this study.

## Author contributions

JL: Conceptualization, Formal analysis, Methodology, Project administration, Software, Supervision, Writing – original draft, Writing – review & editing. JY: Formal analysis, Methodology, Writing – original draft. XG: Funding acquisition, Resources, Writing – original draft. QH: Data curation, Investigation, Methodology, Writing – original draft. YW: Writing – original draft, Data curation, Investigation. QS: Data curation, Investigation, Methodology, Writing – original draft. YuH: Formal analysis, Methodology, Writing – original draft. YX: Data curation, Investigation, Methodology, Writing – original draft. YiH: Funding acquisition, Writing – original draft. LL: Conceptualization, Formal analysis, Methodology, Software, Validation, Writing – review & editing, Supervision.
