# Supplementary material for: Clot patterns determined by DSA and CTA can help predict intracranial atherosclerotic stenosis in acute ischemic stroke patients
Source: Front Neurol. 2024 Jul 24;15:1395764. doi: 10.3389/fneur.2024.1395764 (PMC11303227; doi:10.3389/fneur.2024.1395764)
Supplement: Supplementary file 1 [file Data_Sheet_1.PDF]

1. Flowchart of this study

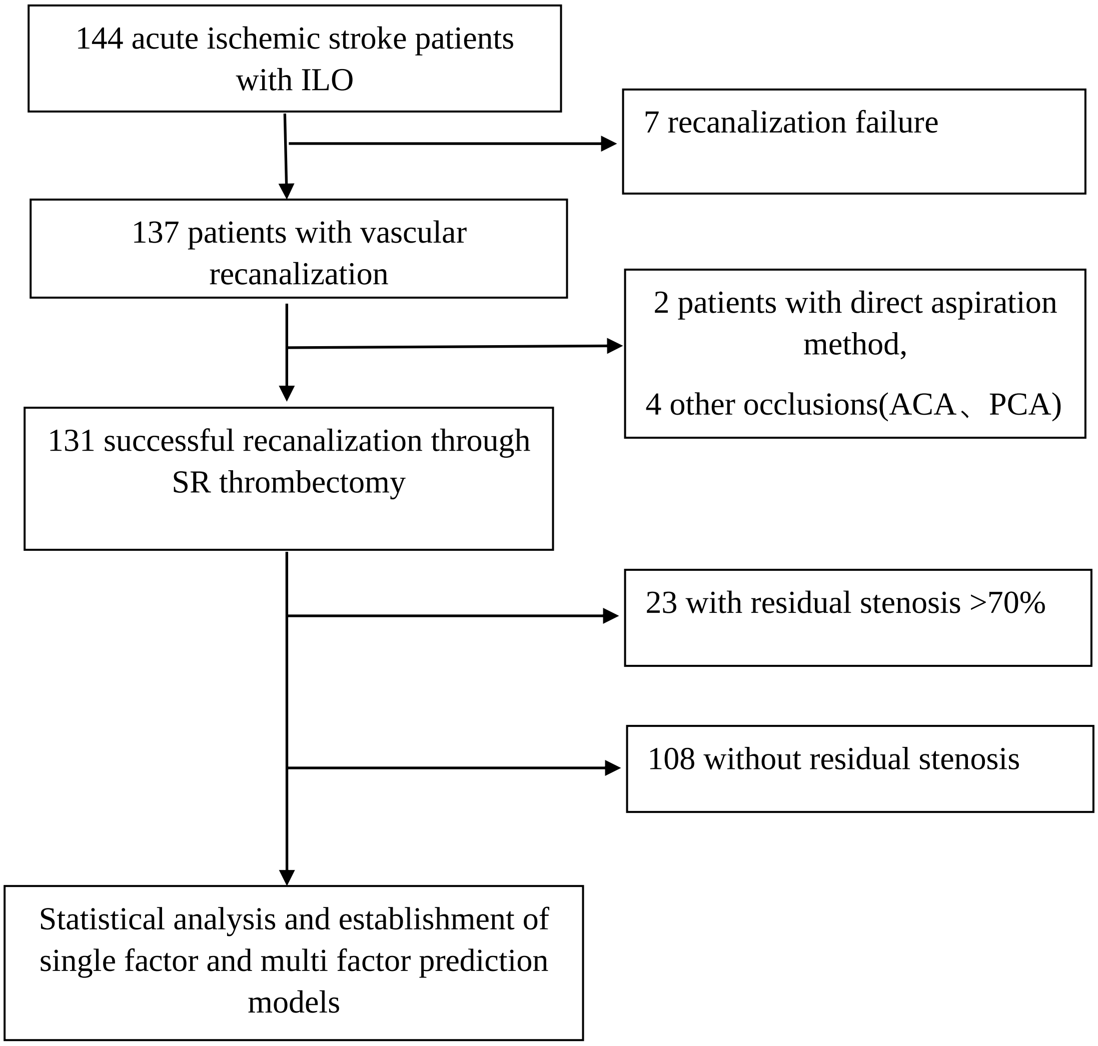

2．dichotomize age, onset to door，The results are as follows:64.5 and 5.3

```
. cutpt residualste0sisafterthrombectm age, near

Empirical cutpoint estimation
Method:                              Nearest to (0,1)
Reference variable:                  residualste0sisafterthrombectm (0=neg, 1=pos)
Classification variable:             age
Empirical optimal cutpoint:          64.5
Sensitivity at cutpoint:             0.57
Specificity at cutpoint:             0.34
Area under ROC curve at cutpoint:    0.45


. cutpt residualste0sisafterthrombectm onsettodoor_hour, near

Empirical cutpoint estimation
Method:                              Nearest to (0,1)
Reference variable:                  residualste0sisafterthrombectm (0=neg, 1=pos)
Classification variable:             onsettodoor_hour
Empirical optimal cutpoint:          5.3083334
Sensitivity at cutpoint:             0.70
Specificity at cutpoint:             0.72
Area under ROC curve at cutpoint:    0.71
```

3．Predicting variables: Clot pattern, LAA, age (>65 vs. <65), onset to door (>5 vs. <5 hours)

or onset to door (>6 vs. <6 hours)

1）Interpretation: for patients with age> 65, presenting >5 hours from stroke onset, toast classified as LAA, and clot topography showing wedge shape, their stroke episodes are most likely caused by ICAS

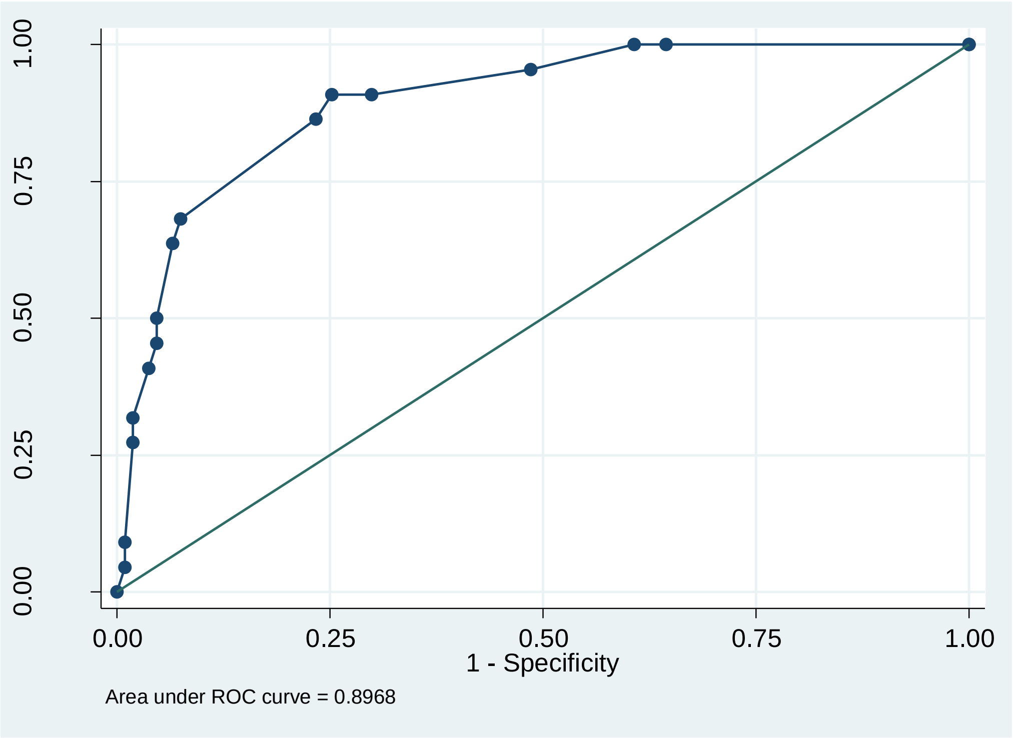

2）Interpretation: for patients with age> 65, presenting >6 hours from stroke onset, toast classified as LAA, and clot pattern showing tapered sign.

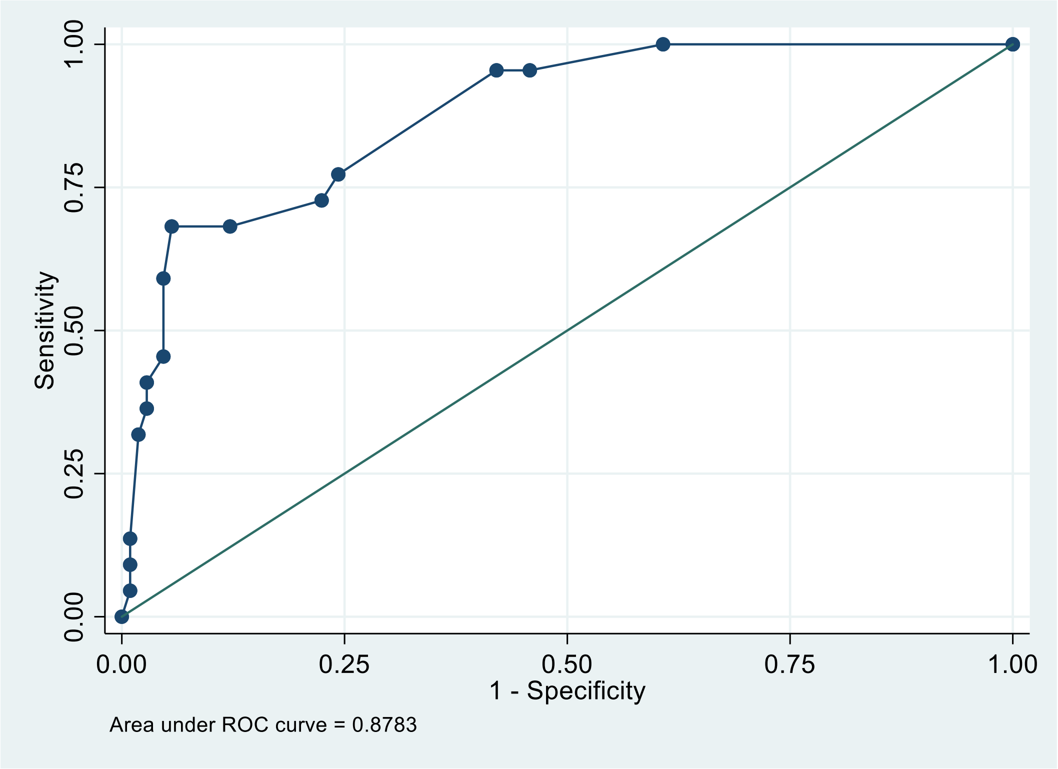

4 . The relationship between various factor and residual stenosis (according to simple logistic regression analysis)

variables	OR	P	95% CI	
LAA	6.27	0.000	2.37	16.63
Clot pattern	20.60	0.000	6.36	66.74
age	0.97	0.063	0.94	1.00
Onset-to-door hour	1.24	0.001	1.10	1.39
smoking	0.65	0.373	0.25	1.68
hypertension	0.83	0.700	0.33	2.09
hypercholesteremia	1.00	0.929	0.98	1.02
diabetes	0.77	0.678	0.23	2.59
occlusion sites	0.80	0.711	0.25	2.57
